# A Modified Arthroscopic Triple‐row Repair Technique for L‐shaped Delaminated Rotator Cuff Tears

**DOI:** 10.1111/os.14039

**Published:** 2024-03-14

**Authors:** Yushun Fang, Shaohua Zhang, Jun Xiong, Qingsong Zhang

**Affiliations:** ^1^ Wuhan Fourth Hospital Wuhan China

**Keywords:** Triple‐row, L‐shaped, Delaminated rotator cuff tears, Separate repair, En masse repair

## Abstract

**Objective:**

To compare the clinical outcomes of a modified arthroscopic triple‐row (TR) repair technique with the suture bridge (SB) repair technique in treating L‐shaped delaminated rotator cuff tears. Various surgical techniques for L‐shaped delaminated rotator cuff tears have been reported, many of which aid in increasing the contact area and pressure of the rotator cuff. However, there is still debate over which technique yields superior results.

**Methods:**

From January 2017 to March 2020, 61 cases of L‐shaped delaminated rotator cuff tears were included in this study. Of these, 34 cases underwent the modified arthroscopic triple‐row repair technique, while 27 cases were addressed with the suture bridge repair technique. Functional assessment was conducted using the American Shoulder and Elbow Surgeons (ASES) score, the University of California Los Angeles (UCLA) shoulder score, the Constant score (CS), and the visual analogue scale (VAS) score. Magnetic Resonance Imaging (MRI) assessments for rotator cuff healing were performed at the 24‐month postoperative mark. Statistical evaluations were conducted using SPSS for Windows (Version 25.0, IBM, Armonk, NY, USA), employing the Wilcoxon signed‐rank test to compare preoperative and postoperative data and ROM differences, and the Mann–Whitney *U* test for statistical differences in clinical outcome scores between the two groups. A *p*‐value of less than 0.05 was considered statistically significant.

**Results:**

Comparative analysis of the preoperative and final follow‐up scores revealed a substantial enhancement in shoulder function, as indicated by the ASES, UCLA, CS, and VAS scores, with statistical significance (*p* < 0.001). At both the preoperative stage and final follow‐up, no notable differences were observed in ASES, UCLA, CS, and VAS scores between the two groups. However, the TR repair group exhibited lower VAS scores than the SB group at 1 and 3 months postoperatively. Active range of motion (ROM) showed significant improvement in both groups. No significant differences in ROM were noted between the two groups either before the surgery or at the final follow‐up.

**Conclusion:**

The study demonstrates that both the modified arthroscopic TR and SB techniques for L‐shaped delaminated cuff tears yield satisfactory outcomes, with no significant differences in overall clinical performance. Notably, early postoperative pain management appears more effective with the modified TR technique, suggesting its potential for enhanced early recovery experiences. This technique's design, promoting securer fixation and optimal contact conditions, is implied to facilitate superior long‐term healing, warranting further investigation into its long‐term benefits.

## Graphical abstract

The study compared the modified arthroscopic triple‐row repair technique and suture bridge repair technique for L‐shaped delaminated rotator cuff tears, finding both methods yielded satisfactory clinical outcomes with no significant difference; however, the modified triple‐row technique offered better pain relief in the early postoperative period and potentially superior long‐term healing benefits. The schematic illustration represents the modified arthroscopic triple‐row repair technique.

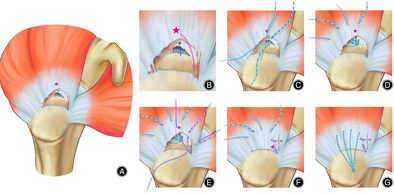

## Introduction

Delamination frequently occurs in cases of rotator cuff tears. Prior studies suggest that its incidence ranges from 38% to 92%.^1^, ^2^, ^3^, ^4^, ^5^, ^6^ Delamination is identified as an adverse prognostic element for the healing of rotator cuffs and is correlated with an increased likelihood of retears.^2^, ^3^, ^7^, ^8^ Various surgical techniques have been reported for the treatment of delaminated rotator cuff tears, such as double‐row technique (separate double‐row and lamina‐specific double‐row), suture bridge technique (e.g., medially knotted bridge, knotless bridge, layered transosseous‐equivalent, en masse suture bridging, dual‐layer suture bridging, and double‐layer lasso‐loop), bursal layer only repair technique, and arthroscopically assisted mini open technique, and so on.^9^, ^10^, ^11^, ^12^, ^13^, ^14^, ^15^, ^16^, ^17^, ^18^ These techniques can be divided into en masse repair and separate repair. En masse repair may result in inevitable tension mismatch which is caused by tying torn layers together. Separate repair could reduce the subjective pain intensity of the patient by avoiding the tension mismatch.^19^

However, the retraction orientation of layers differs in L‐shaped rotator cuff tears. The traditional double‐row repair technique may fail to adequately address the retraction at the inflection point of the “L,” potentially compromising the coverage of the repaired rotator cuff on the footprint. A modified triple‐row technique we used for L‐shaped delaminated cuff tears belongs to the separate repair technique category, which can balance the tension distribution and the direction of the cuff reduction, and obtain good coverage to the footprint on the greater tuberosity. In our study, we aim to explore a surgical approach that optimally increases the contact area and pressure on the rotator cuff while ensuring a superior tension match. This endeavor seeks to identify a technique that not only addresses the structural integrity of the rotator cuff but also enhances the biomechanical environment conducive to healing and functional recovery.

## Methods

This is a retrospective, single‐center, non‐randomized case–control study. The study protocol was approved by the Institutional Review Board of Wuhan Fourth Hospital, Hubei Province, China. (Reference number, KY2022‐079‐01).

### Patient Selection

The inclusion criteria for this retrospective study were: (i) medium to large L‐shaped delaminated rotator cuff tears that had been repaired with the modified triple‐row technique or suture bridge technique; and (ii) follow‐up for at least 24 months. The exclusion criteria included: (i) massive or partial‐thickness rotator cuff tears; (ii) other repair techniques; and (iii) revision surgery. After application of these criteria, a total of 61 patients who underwent arthroscopic rotator cuff repair were included in this study. Indication and contraindications are summarized in Table 1.

**TABLE 1:** Indications and contraindications for modified arthroscopic triple‐row technique

Indication
Medium to large L‐shaped delaminated rotator cuff tears
Contraindications
1. Massive rotator cuff tears. 2. Partial‐thickness rotator cuff tears. 3. Very osteoporotic bone quality. 4. Very poor tissue quality.

### Clinical Evaluation

For the functional evaluation of the shoulder, various scoring systems were employed including the American Shoulder and Elbow Surgeons (ASES) score, the University of California Los Angeles (UCLA) shoulder score, the Constant score (CS), and the visual analogue scale (VAS) score. Additionally, to assess shoulder function both preoperatively and during the last follow‐up, active range of motion (ROM) was measured, encompassing forward elevation (FE), external rotation (ER) at the side, and internal rotation (IR) at the back. For the categorization of internal rotation on the back, the classification system suggested by Ide *et al*.^20^ was utilized, converting the values into consecutive numbered levels (T1 to T12 as 1 to 12, L1 to L5 as 13 to 17, sacrum as 18, and buttock as 19).

Preoperative MRI scans showed the pattern and size of the tears, and the presence of delamination. Postoperative MRI scans were performed as radiological assessments to determine tendon integrity or re‐tear in all patients. During the surgical procedure, the specific pattern of the delaminated rotator cuff tears and the retraction direction of the delaminated layers were evaluated through direct arthroscopic examination.

### Surgical Technique

#### Patient Preparation and Arthroscopic Evaluation

The surgical intervention was performed under general anesthesia, complemented with an interscalene block for improved analgesia. The patient was positioned in the lateral decubitus position, with the affected extremity suspended and subjected to axial traction using a weight ranging from 3 to 5 kg. Four portals were used in the surgical procedure: posterior, anterior, anterolateral, and posterolateral. Extra portals were created as needed to ensure the optimal angle for suture anchor placement.

A comprehensive diagnostic arthroscopy was conducted to identify and address any intra‐articular pathologies. Detailed evaluation of the torn tendon's status and mobility, retraction extent, and the presence and pattern of delamination was carried out *via* the anterolateral or posterolateral portal within the subacromial space. This was followed by a standard anterior acromioplasty.

#### Repair of the Articular Layer

The articular layer was grasped and evaluated. Soft tissue releases and mobilization of the rotator cuff were conducted when deemed essential. A medialized repair was applied if the articular layer still did not reach the medial edge of the greater tuberosity.

**FIGURE 1:**
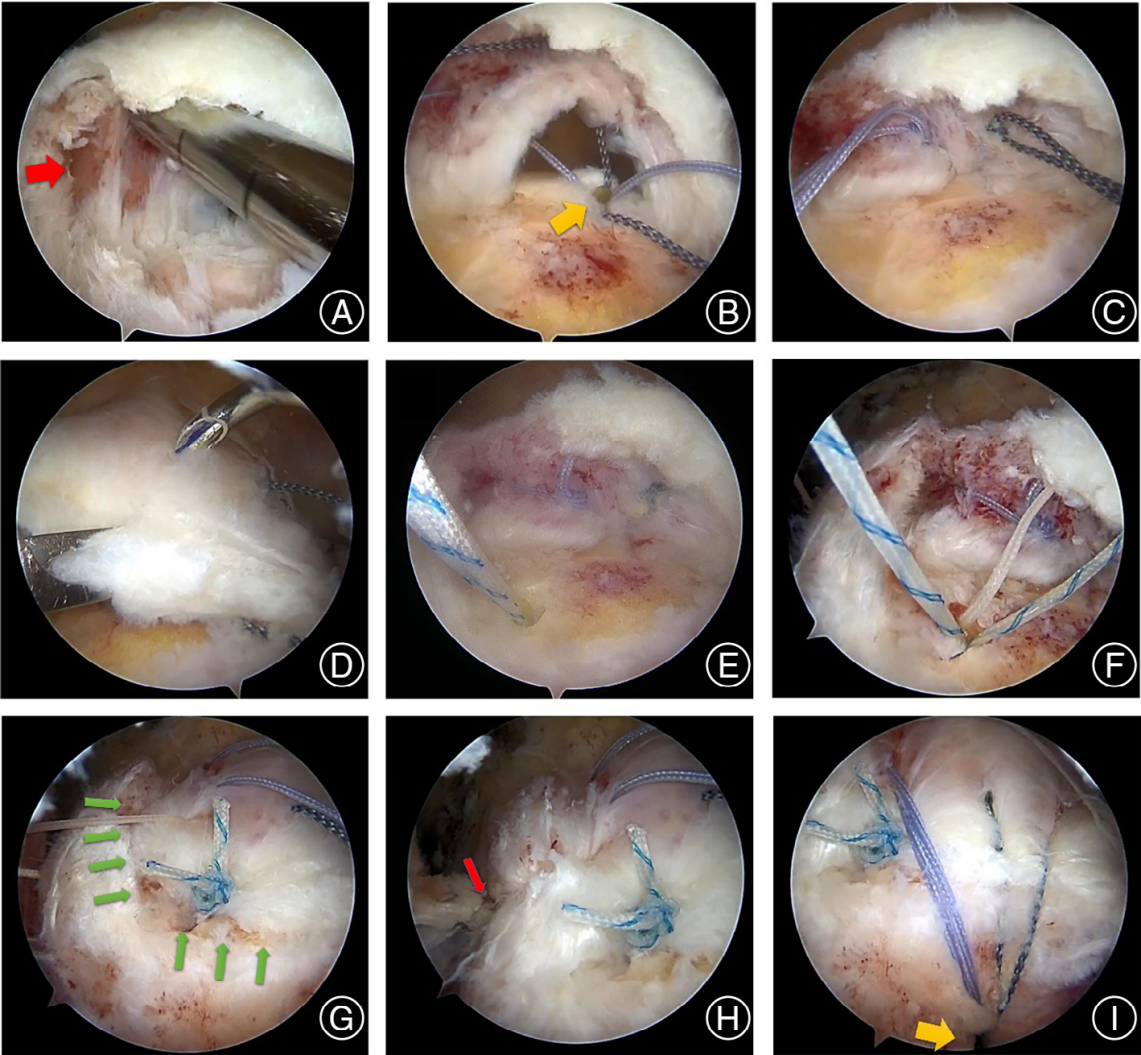
A left shoulder with L‐shaped delaminated rotator cuff tears that underwent a modified triple‐row fixation, as viewed from the posterolateral portal. (A) Debridement of the split surfaces of delaminated tears was performed. The red arrow shows the synovial‐like lining between the two layers. (B) The medial row anchor was positioned at the greater tuberosity's medial edge, as indicated by the yellow arrow. The sutures from the medial row were then arranged through the articular layer in a mattress‐like configuration. (C) For the repair of the articular layer, three non‐sliding surgical knots were utilized. The suture limbs were kept intact (not cut) to facilitate the repair of the bursal layer. (D) The repair of bursal layer was performed in reducing state of the cuff to obtain better tension match and more contact area. (E) The middle‐row anchor was inserted on the lateral edge of the greater tuberosity (yellow arrow) where could achieve the bursal layer anatomic repair. (F) Suture distribution of the middle‐row anchor. (G) The bursal layer was reduced by one suture of middle‐row anchor. The green arrows show the L‐shaped tear of the bursal layer. (H) A side‐to‐side technique was performed to achieve margin convergence of the longitudinal tear in the L‐shaped tear with the other suture of middle‐row anchor (red arrow). (I) The lateral‐row anchor (yellow arrow) was placed to improve the contact pressure.

Debridement was performed on the footprint of the greater tuberosity and the split surfaces of the delaminated tears (Figure 1A). Greater tuberosity decortication or microfracture might improve the healing potentials of the tendon to the bone.

Based on the tear's size, either one or two suture anchors were placed at the medial edge of the greater tuberosity to facilitate the repair of the articular layer. Subsequently, the sutures from the anchors in the medial row were threaded through the articular layer utilizing a suture passing instrument, as depicted in Figure 1B. Three non‐sliding surgical knots were used in this procedure to avoid the influences of healing of delamination due to large knots. Suture limbs were left intact (without cutting) for the repairing of the bursal layer (Figure 1C).

#### Repair of the Bursal Layer

The key point of the bursal layer retraction was evaluated by the grasper on the torn edge. The repair of the bursal layer was performed with suture limbs of the medial‐row anchors by pulling the bursal layer with the tendon grasper to make it reduced, so that the cuffs could be repaired with minimal tension (Figure 1D). Each time a suture limb was passed, it was subsequently retrieved through the anterior portal using a grasper.

A middle‐row double‐loaded anchor was inserted at the lateral margin of the rotator cuff footprint where could anatomically reduce the bursal layer by the key point of cuff retraction (Figure 1E). The bursal layer was reduced by a simple repair technique with one suture of the double‐loaded anchor (Figure 1F, 1G). A side‐to‐side technique was performed to repair the longitudinal tear in the L‐shaped tear with the other suture (Figure 1H). Each suture tail from the middle‐row anchor was cut down.

Every suture limb from the medial‐row anchors, after being threaded through the bursal layer of the cuff, was secured using a knotless lateral‐row anchor. This anchor was subsequently positioned about 10 mm distal to the greater tuberosity's lateral edge and slightly posterior to the bicipital groove (Figure 1I). Applying suitable tension to the suture limbs was crucial during the insertion of the anchor.

**FIGURE 2:**
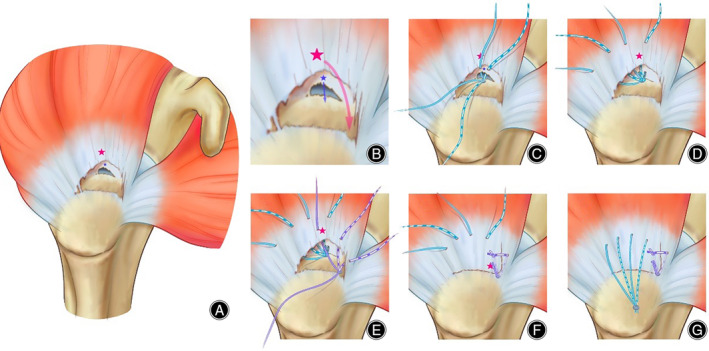
Illustration of the modified triple‐row technique for the L‐shaped delaminated rotator cuff tears (right shoulder). (A) The overall view of delaminated rotator cuff tears (red star and blue star show the key points of the two layers retraction). (B) The retraction directions are different in the articular layer (blue star and blue arrow) and the bursal layer (red star and red arrow). (C) The position and suture distribution of the medial‐row anchor. (D) Suture distribution on the bursal layer after the repair of the articular layer. (E) The position and the suture distribution of the middle‐row anchor. (F) The reducing of the bursal layer and the margin convergence of the longitudinal tear was achieved by the middle‐row anchor. (G) The lateral‐row anchor was positioned distally, relative to the greater tuberosity's lateral edge.

The pearls and pitfalls are shown in Table 2. The illustration of the Triple‐row technique details is shown in Figure 2.

**TABLE 2:** Pearls and pitfalls

Pearls
1. Anatomic repair (the bursal layer and the articular layer of the rotator cuff are restored to their native insertion). 2. The tension match is better due to the separate reducing and separate repair. 3. The contact area of rotator cuff and footprint is maximized. 4. The contact pressure of the layer‐to‐layer and layers‐to‐footprint are improved by lateral row anchor. 5. The healing of delamination is improved by debridement of the synovial‐like lining. 6. Does not increase the use of anchors.

Pitfalls
1. It is important to evaluate the presence and pattern of delamination through lateral portal (including anterolateral and posterolateral portal). 2. To minimize the knots in the articular layer repair to avoid the influences of healing of the layers. 3. Excessive decortication of the footprint should be avoided when preparing the greater tuberosity to allow for optimal anchor stability. 4. Suture distribution of the bursal layer should be designed by reducing it with a grasper.

#### Postoperative Care

After the surgery, all patients were immobilized using a shoulder brace for a duration of six weeks. Starting from the first day following the operation, passive range of motion (ROM) exercises and table slides were implemented. Active ROM and proprioceptive training were introduced six weeks later. The initiation of rotator cuff strengthening exercises, based on the quality of the tendon and the presence of osteoporosis, was recommended to be delayed until at least 12–16 weeks postoperative to minimize the risk of re‐tear.

#### Follow‐up

In this study, comprehensive follow‐up evaluations were meticulously conducted to assess the effectiveness of the modified arthroscopic triple‐row repair technique for L‐shaped delaminated rotator cuff tears. Functional outcomes were evaluated using a range of validated scoring systems, including the ASES score, the UCLA shoulder score, and the CS, at predefined intervals post‐surgery: preoperatively, at 3, 6, 12, and 24 months. Pain levels were quantitatively assessed using the VAS score at baseline and postoperatively at 1, 3, 6, 12, and 24 months to monitor the progression of pain management over time. Additionally, ROM of the shoulder joint was assessed preoperatively and postoperatively at 3, 6, 12, and 24 months to evaluate functional recovery. MRI was utilized to assess the integrity and healing of the rotator cuff preoperatively and at 6 and 24 months post‐operation. This follow‐up protocol was designed to ensure a thorough evaluation of the surgical outcomes, providing a comprehensive overview of the functional recovery, pain management, and rotator cuff healing following the modified arthroscopic triple‐row repair technique.

### Statistical Analysis

Statistical evaluations were conducted utilizing SPSS for Windows (Version 25.0, IBM, Armonk, NY, USA). To compare the aggregated preoperative and postoperative data, as well as differences in ROM, the Wilcoxon signed‐rank test was applied. For evaluating statistical variances in clinical outcome scores between the two groups, the Mann–Whitney U test was employed. A *p*‐value of less than 0.05 was considered the threshold for statistical significance.

## Results

Between January 2017 and March 2020, 61 patients with L‐shaped delaminated rotator cuff tears underwent an arthroscopic rotator cuff repair using modified triple‐row technique (34 cases) and suture bridge technique (27 cases). No notable variances were observed between the two groups in terms of the patient's age at the time of surgery, gender, or the length of the postoperative follow‐up period. The characteristics of the patients are concisely presented in Table 3.

**TABLE 3:** Patient demographics

	Triple‐row (*n* = 34)	Suture bridge (*n* = 27)	*p* value
Average age (years)	57.68 ± 7.40	60.33 ± 6.42	0.1518
Male: female	19:15	15:12	0.1777
Duration of symptoms (months)	6.74 ± 3.24	5.89 ± 2.15	0.1275
Tear size			
Medium (1–3 cm on MRI)	11	8	0.1608
Large (3–5 cm on MRI)	23	19	
Type of tear			
Traumatic	3	2	0.1637
Degenerative	31	25	
Follow‐up (months)	24.15 ± 3.02	24.20 ± 3.62	0.4740

*Note*: Continuous variables are presented as mean ± standard deviation.

### Assessment of Functional Scores and Pain Levels

The ASES, UCLA, CS, and VAS scores showed significant improvements in shoulder function, as evidenced by the comparison between preoperative scores and those documented at the final follow‐up, with statistical significance (*p* < 0.001). No significant differences in these scores were observed between the two groups, either preoperatively or at the final follow‐up, as detailed in Table 4. Notably, the triple‐row (TR) group exhibited lower scores in comparison to the suture bridge (SB) group at the 1st and 3rd months postoperatively, as elaborated in Table 5.

**TABLE 4:** Comparison of clinical results

	Preoperative		Final follow‐up		*p* value
	mean ± SD (range)	*p* value	mean ± SD (range)	*p* value	
ASES score (max. 100)					
Group TR*	36.44 ± 13.68	0.0867	93.09 ± 6.55	0.1427	<0.0001^‡^
Group SB^†^	42.70 ± 13.76		90.26 ± 8.08		<0.0001^‡^
UCLA shoulder score (max. 35)					
Group TR*	12.88 ± 4.51	0.1158	32.56 ± 4.26	0.3758	<0.0001^‡^
Group SB^†^	14.81 ± 4.75		31.59 ± 3.96		<0.0001^‡^
CS (Max. 100)					
Group TR*	39.32 ± 15.24	0.1468	84.85 ± 12.42	0.4236	<0.0001^‡^
Group SB^†^	45.48 ± 16.87		82.22 ± 12.49		<0.0001^‡^
VAS score (max. 10)					
Group TR*	5.82 ± 0.98	0.2203	0.94 ± 0.84	0.3411	<0.0001^‡^
Group SB^†^	5.48 ± 1.13		1.15 ± 0.80		<0.0001^‡^

* TR, Triple‐row.

^†^ SB, Suture bridge.

^‡^ Statistically significant difference between pre‐ and postoperative values (*p* < 0.05).

**TABLE 5:** Comparison of VAS score between two groups

	Group TR* (mean ± SD)	Group SB^†^ (mean ± SD)	*p* value
Preoperative	5.82 ± 0.98	5.48 ± 1.13	0.2203
1 month postoperatively	3.14 ± 1.00	4.07 ± 0.90	0.0005
3 months postoperatively	2.03 ± 0.71	2.63 ± 0.55	0.0007
6 months postoperatively	1.88 ± 0.72	2.15 ± 0.70	0.1597
12 months postoperatively	0.94 ± 0.84	1.15 ± 0.80	0.3411
Final follow‐up	0.18 ± 0.38	0.15 ± 0.36	0.7713

* TR, Triple‐row.

^†^ SB, Suture bridge.

### Evaluation of ROM

Significant improvements in active ROM, including anterior elevation, abduction, external rotation, and internal rotation, were observed in both the modified triple‐row TR and SB technique groups. These enhancements, from preoperative assessments to the final follow‐up, indicate effective restoration of shoulder mobility post‐surgery. Importantly, there were no substantial differences in ROM outcomes between the TR and SB groups, affirming the comparability of both surgical techniques in facilitating functional recovery, as comprehensively presented in Table 6.

**TABLE 6:** Preoperative versus postoperative active range of motion

	Preoperative		Final follow‐up		*p* value
	Mean ± SD (range)	*p* value	Mean ± SD (range)	*p* value	
Anterior elevation, deg					
Group TR*	100.15 ± 20.60	0.5132	166.32 ± 12.02	0.8130	<0.0001^‡^
Group SB^†^	103.15 ± 12.26		165.56 ± 12.72		<0.0001^‡^
Abduction, deg					
Group TR*	94.56 ± 18.41	0.3298	163.53 ± 14.27	0.3792	<0.0001^‡^
Group SB^†^	98.70 ± 12.59		160.18 ± 14.56		<0.0001^‡^
External rotation, deg					
Group TR*	32.50 ± 11.84	0.5551	49.41 ± 10.83	0.6693	<0.0001^‡^
Group SB^†^	34.44 ± 13.28		50.56 ± 9.26		<0.0001^‡^
Internal rotation^§^					
Group TR*	16.09 ± 1.88	0.2248	12.38 ± 1.88	0.1255	<0.0001^‡^
Group SB^†^	15.52 ± 1.62		13.07 ± 1.44		<0.0001^‡^

* TR Triple‐row.

^†^ SB Suture bridge.

^‡^ Statistically significant difference between pre‐ and postoperative values (*p* < 0.05).

^§^ Values of internal rotation were converted into contiguously numbered groups as proposed by Ide *et al*.^11^

### Preoperative and Postoperative MRI Evaluation

**FIGURE 3:**
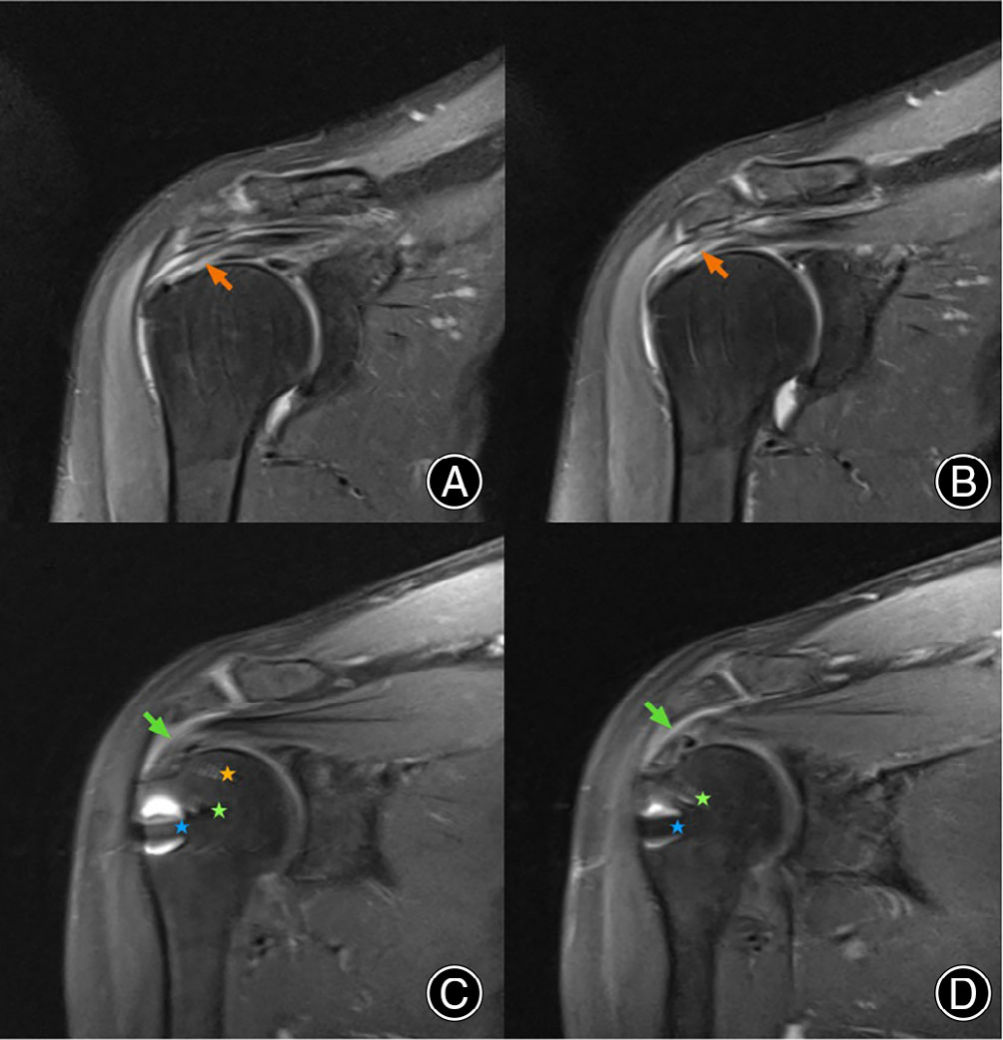
This figure presents preoperative and postoperative MRI (T2 coronal view) images following the application of the modified arthroscopic triple‐row repair technique for an L‐shaped delaminated rotator cuff tear. Images A and B are preoperative MRIs highlighting the delamination of the rotator cuff, indicated by orange arrows. Images C and D represent MRI scans taken six months postoperatively, where green arrows point to significant healing at the bone interface of the rotator cuff and the greater tuberosity footprint area, as well as the disappearance of delamination. The orange star marks the location of the medial‐row anchor, the green star indicates the middle‐row anchor, and the blue star identifies the lateral‐row anchor. It is noted that the high signal area surrounding the lateral‐row anchor is due to artifact produced by the metal sleeve within the anchor.

Following the arthroscopic repair of L‐shaped delaminated rotator cuff tears using both techniques, MRI assessments were systematically performed preoperatively and at the six‐month postoperative mark. The T2 coronal MRI evaluations revealed that both repair methods resulted in significant healing at the bone interface of the rotator cuff and greater tuberosity footprint area. Notably, the scans demonstrated the resolution of delamination across cases treated with either technique. (Figure 3).

### Complications

In the course of postoperative follow‐up, some patients exhibited an unexpected poor recovery of ROM at the 3‐month evaluation—this involved one individual from the modified TR group and two from the SB group. Subsequent to the identification of these ROM recovery issues, a focused rehabilitation regimen was initiated, covering a duration from 1 to 3 months, which effectively facilitated the gradual normalization of shoulder mobility. This tailored rehabilitative approach underlines the essential role of proactive and individualized rehabilitation in overcoming challenges in ROM recovery post‐surgery and achieving satisfactory functional restoration.

## Discussion

In this retrospective, non‐randomized case‐control study, patients from both groups who underwent arthroscopic rotator cuff repair, either *via* the modified triple‐row technique or the suture bridge technique, showed improved outcomes. Differences in VAS scores between the two groups were observed at 1 and 3 months post‐surgery.

Delamination is characterized as a horizontal split within the layers of the rotator cuff.^21^ In cases of delaminated rotator cuff tears, it is typical to distinguish between the two principal layers: the outer bursal side and the inner articular side.^10^, ^22^ It is considered to be caused by a variety of possible factors, such as the dissimilar stress and extent of retraction between the bursal and articular layers,^6^, ^13^ different collagen orientations between these two layers,^6^ and degenerative changes within the tendon.^3^, ^5^, ^23^ MacDougal and Todhunter^3^ found that the articular layer is typically more retracted than the bursal layer in delaminated rotator cuff tears. Slagmolen *et al*.^24^ asserted that there are differences in strain and displacement between the two layers during active shoulder motion. Kim *et al*.^13^ observed that, under loading conditions, the tension experienced within the rotator cuff is relatively greater in the articular layer as compared to the bursal layer. Hence, repairing layers together inevitably leads to tension mismatches, potentially increasing patients' subjective pain intensity. Biomechanical studies have additionally demonstrated that separate repair strategies can closely replicate natural biomechanics, thereby potentially lowering the risk of rotator cuff repair failures.^17^ To avoid the tension mismatch and the risk of repair failure, separate repair is recommended.^17^, ^19^, ^24^, ^25^, ^26^ A meta‐analysis also revealed that for patients with delaminated rotator cuff tears, separate repair offers better outcomes in both Constant scores and shoulder external rotation compared to an en masse repair approach.^27^

### Identifying and Managing Delamination

**FIGURE 4:**
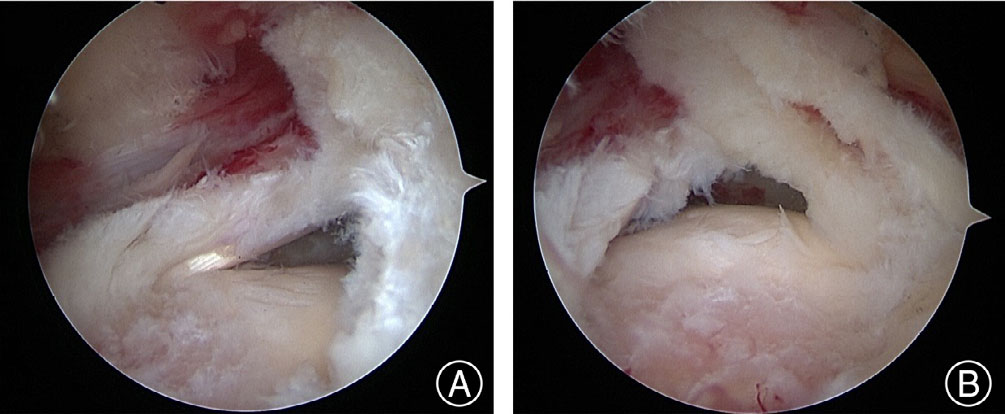
A left shoulder with delaminated rotator cuff tears. (A) The delamination was hidden when viewing from the posterior portal. (B) The posterolateral viewing portal could provide an en face view which improved the identification of delamination.

Identifying delamination is crucial before proceeding with the repair of rotator cuff tears. We chose posterolateral portal as a routine viewing portal to evaluate the presence and pattern of delamination. Moezzi^28^ suggested that delamination might often be missed unless it is deliberately searched for from a more lateral portal. Han *et al*.^2^ found that the posterolateral and lateral portals offer a direct view of the rotator cuff tear. They recommended employing differential portals and rotating the arm to achieve an optimal perspective for viewing and manipulating the rotator cuff directly. This approach includes examining the undersurface, anterior and posterior margins, delaminated layers, and the footprint. Figure 4 shows the difference of the viewing from posterior portal and posterolateral portal when identifying delaminated rotator cuff tears. Therefore, posterior delamination will be hidden and left untreated if surgeons only view from the posterior portal.

It is also important to debride the surface of the delamination cleavage. Sonnabend *et al*.^6^ identified evidence of a synovial‐like lining between the two layers, and this is believed to play a role in preserving the lamination and potentially hindering its repair. It means that if the tendon is repaired without first removing the synovial‐like lining, it may continue to exist as an intratendinous tear within the rotator cuff. Therefore, it is recommended that debridement of the delamination cleavage should be performed before repairing to promote the mutual healing of the layers.^29^

### Articular Layer Repair

Medial‐row anchors are placed at the medial edge of the footprint on the greater tuberosity where the superior capsule originally inserted to perform a separate repair of articular layer. A crucial aspect is repairing the articular layer without tension. A soft tissue release, rotator cuff mobilization, or medialized repair would be performed as necessary. Three nonsliding surgical knots are used to repair the articular layer. The small knots between the two layers promote increased interlayer contact which enhances the healing process. The suture limbs which are used to repair the articular layer are left intact for the bursal layer repairing.

### L‐shaped Tear Repair

Davidson and Burkhart^30^ proposed a geometric classification for rotator cuff tears, in which a longitudinal tear was described as Type 2, including L‐shaped and U‐shaped tears. Sallay *et al*.^31^ described L‐shaped and reverse L‐shaped tears as comprising both transverse and longitudinal elements, which constitute around 30% of all full‐thickness posterosuperior tears. A key feature of the L‐shaped tears, which typically extend along the interval between the supraspinatus and infraspinatus, or the reverse L‐shaped tears, which often propagate through the rotator interval, is that their free margins are either taut or lax. This results in one edge being more mobile compared to the other, which is different form U‐shaped tear.^32^ L‐shaped tears are similar to reverse L‐shaped tears in configuration but differ in direction of reducing. Thus, L‐shaped and reverse L‐shaped tears may be considered a single pattern.

Sano *et al*.^33^ in a cadaveric study found that there was a high concentration of stress observed at both the posterior tendon edge and the base of the longitudinal tear in L‐shaped tears. There is a tendency to retract on the key point of cuff reduction by this stress distribution pattern in L‐shaped tears. General repair techniques such as double‐row technique or suture bridge technique can be difficult to reconstruct the cuff anatomically. The anatomical positioning of the repaired cuff may be significantly altered, frequently being drawn medially and posteriorly.^34^ The rotator cuff footprint contact area is limited due to the retraction. Therefore, the key point of the L‐shaped rotator cuff tear reduction must be evaluated carefully by the grasper on the torn edge. The sutures distribution of the bursal layer should then be designed by reducing the key point of L‐shaped tears to the native footprint, which would make the tension of the bursal layer match better and increase the contact area.

### Middle‐row Anchor Technique

After all the suture limbs of medial‐row anchors are passed through the bursal layer, a middle‐row anchor is inserted at the corresponding point on the lateral edge of the greater tuberosity where could achieve anatomic repair of the bursal layer. The middle‐row anchor could not only restore the bursal layer to its footprint, but also achieve margin convergence of the longitudinal tear in L‐shaped tears by the side‐to‐side technique. It maximizes the contact area of the cuff and its footprint. Once the bursal layer is reduced to the tuberosity by the middle‐row anchor, visualization is better.^35^ It should be noted that excessive decortication of the footprint should be avoided when preparing the greater tuberosity to allow for optimal anchor stability.^34^ In particular, the reservation of the cortical bone is more important for middle‐row anchor.

### Triple‐row Technique Advantages

According to Park *et al*.,^36^, ^37^, ^38^ several factors are considered crucial in enhancing the healing potential of cuff repairs. These include more secure fixation, an enlarged contact area, and increased pressure between the rotator cuff tendon and the tuberosity. Ostrander and McKinney^39^ described a triple‐row technique that demonstrated a notably increased contact area and contact pressure within the rotator cuff footprint compared to standard suture bridge and double‐row techniques. In fact, several triple‐row techniques have been documented to enhance the contact area and pressure within the cuff footprint. This is achieved by incorporating an additional middle‐row anchor to secure the leading edge of the cuff to the bone.^34^, ^35^, ^40^, ^41^, ^42^, ^43^ In contrast, en masse repair techniques disregard the multilayered structure of the rotator cuff and neglect to restore the native superior capsule and cuff insertion.

Unlike traditional triple‐row techniques, our technique performed a separate repair by medial‐row and middle‐row anchors to improve the rotator cuff footprint contact area and the tension match. Less tension may have reduced early postoperative pain, which causes lower VAS scores at 1 and 3 months postoperatively in the triple‐row group of our study. The pressure of the rotator cuff tendon and tuberosity is improved by lateral‐row anchor. Actually, only one lateral‐row anchor is typically enough in this technique as the layers of the cuff are reduced by middle‐row and medial‐row anchors. Therefore, this technique does not increase the use of anchors compared to the double‐row technique or suture bridge technique. Furthermore, it is also a modification of our technique which performs a margin convergence with middle‐row anchor to repair the longitudinal tear in the L‐shaped tears.

## Limitations

Our study acknowledges certain limitations that merit consideration. First, the retrospective design of our investigation, while providing valuable insights, may introduce inherent biases associated with the retrospective analysis. This design limitation restricts our capacity to establish causation definitively and may affect the generalizability of our findings.

Second, the study's scope, constrained by a relatively limited patient cohort, potentially affects the statistical power and robustness of our conclusions. The size of our sample, although sufficient for preliminary observations, might not fully capture the variability and complexity of clinical outcomes associated with the modified arthroscopic triple‐row repair technique.

Third, the innovative nature of our modified triple‐row technique, while promising, necessitates further validation through comprehensive biomechanical studies. Future research, ideally incorporating prospective designs and larger patient samples, is essential to substantiate the biomechanical advantages and clinical efficacy of this technique over traditional methods.

By addressing these limitations, future studies can build on our work to offer deeper insights and more definitive conclusions regarding the optimal surgical approach for L‐shaped delaminated rotator cuff tears.

## Conclusion

Both modified triple‐row technique and suture bridge technique used for the treatment of L‐shaped delaminated cuff tears achieved satisfactory clinical outcomes that do not differ significantly. Patients treated with the modified triple‐row technique showed better pain experience compared with those repaired with the suture bridge technique within 3 months postoperatively. This may be related to the better tension match created by the modified triple‐row technique. It is speculated that the securer fixation, larger contact area, and more suitable contact pressure created by the modified triple‐row technique allows better healing in the long term follow‐up.

## Author Contributions

Yushun Fang, was primarily responsible for manuscript writing and performing the surgical procedures. Qingsong Zhang, mainly focused on the surgical operations. Shaohua Zhang and Jun Xiong, were chiefly in charge of data collection and analysis.

## Ethics Statement

This study was conducted in accordance with the ethical standards and approval of the Wuhan Fourth Hospital's Ethics Committee. The research protocol was reviewed and approved, ensuring compliance with the principles laid out in the Declaration of Helsinki. All participants provided informed consent prior to their inclusion in the study.
